# Loss of Genome Fidelity: Beta HPVs and the DNA Damage Response

**DOI:** 10.3389/fmicb.2017.02250

**Published:** 2017-11-15

**Authors:** Sebastian O. Wendel, Nicholas A. Wallace

**Affiliations:** Division of Biology, Kansas State University, Manhattan, KS, United States

**Keywords:** beta HPV, DNA-damage response, skin cancer, genomic fidelity, tumorigenesis, epidermodysplasia verruciformis, viral oncogenesis, p300

## Abstract

While the role of genus alpha human papillomaviruses in the tumorigenesis and tumor maintenance of anogenital and oropharyngeal cancers is well-established, the role of genus beta human papilloviruses (β-HPVs) in non-melanoma skin cancers (NMSCs) is less certain. Persistent β-HPV infections cause NMSCs in sun-exposed skin of people with a rare genetic disorder, epidermodysplasia verruciformis. However, β-HPV infections in people without epidermodysplasia verruciformis are typically transient. Further, β-HPV gene expression is not necessary for tumor maintenance in the general population as on average there is fewer than one copy of the β-HPV genome per cell in NMSC tumor biopsies. Cell culture, epidemiological, and mouse model experiments support a role for β-HPV infections in the initiation of NMSCs through a “hit and run” mechanism. The virus is hypothesized to act as a cofactor, augmenting the genome destabilizing effects of UV. Supporting this idea, two β-HPV proteins (β-HPV E6 and E7) disrupt the cellular response to UV exposure and other genome destabilizing events by abrogating DNA repair and deregulating cell cycle progression. The aberrant damage response increases the likelihood of oncogenic mutations capable of driving tumorigenesis independent of a sustained β-HPV infection or continued viral protein expression. This review summarizes what is currently known about the deleterious effects of β-HPV on genome maintenance in the context of the virus's putative role in NMSC initiation.

## Introduction

Human papillomavirus (HPV) is a family of small, non-enveloped double-stranded DNA viruses that infect mucosal and cutaneous epithelia. This family is comprised of five genera (alpha, beta, gamma, nu, and mu) spanning across over 396 different HPV types potentially inhabiting human skin (Bzhalava et al., 2014). Classification of the HPV family is based on the sequence of the L1 capsid protein (Bernard et al., 2010; de Villiers, 2013). The alpha and beta genera are most widely studied because of the pathogenesis associated with some members of these genera. α-HPV causes genital warts (low risk types, HPV 6, and 11 for example) and genital cancer (high risk types, HPV 16, 18, 31, and 45 for example) (Tommasino, 2014; Doorbar et al., 2015). While β-HPV is known to cause flat warts and non-melanoma skin cancer in individuals with compromised immune systems (Cubie, 2013), there is a growing interest in determining if these viruses can cause tumors in the general population. Further, while so called “high risk” α-HPVs have been identified, the relative oncogenic potential among the beta genus of HPV is still being discussed. Moreover, despite already consisting of ~50 types of HPV that are sub-classified in five species (Van Doorslaer, 2013), it is likely that a large portion of β-HPV types have yet to be discovered (Ekström et al., 2013; Bzhalava et al., 2014, 2015). Because the first β-HPV types isolated were HPV 5 and HPV 8 from cutaneous squamous cell carcinomas (cSCC) of patients with the rare disorder Epidermodysplasia Verruciformis (EV) (Pfister et al., 1981; Kremsdorf et al., 1982, 1983), these two β-HPVs have been the most extensively examined.

Because β-HPVs do not generally persist in tumors, they are hypothesized to act through a “hit and run” mechanism (Pfister, 2003; Hufbauer and Akgül, 2017). Specifically, β-HPVs are believed to hinder the repair of DNA damage caused by UV radiation making mutations more likely. For a virus that infects an area frequently exposed to UV radiation and yet requires proliferating cells to complete its lifecycle, it is reasonable that β-HPVs have developed mechanisms to prevent the cell cycle arrest that accompanies repair of UV damaged DNA. However, this could have severe consequences for the host cell as the UV-induced mutations will remain after the viral infection is cleared and could drive the development of non-melanoma skin cancers (NMSCs) without continued expression of β-HPV genes. In this review, we will begin by providing a brief synapsis of the evidence that β-HPV infections cause NMSCs as well as the cellular signaling pathways that respond to UV insults. We will provide a concise discussion of α-HPV associated tumorigenesis, as these more clearly defined mechanisms of transformation provide a useful and relevant comparison for β-HPV associated oncogenesis. Then, we will move on to the *in vitro* and *in vivo* data that demonstrate the ability of β-HPV genes to disrupt the repair of DNA, destabilizing the host genome in a manner consistent with a “hit and run” mechanism of tumorigenesis.

### Transformation by alpha genus HPV oncogenes

Extensive evidence has established α-HPV as the causative agent in cervical cancers and in many other malignancies in the oropharynx and throughout the anogenital tract (Hobbs et al., 2006; Moody and Laimins, 2010; Carter et al., 2011; D'Souza and Dempsey, 2011; Tommasino, 2014). Infections with high risk α-HPVs can begin a multi-decade process where cells are immortalized, accumulate destabilized genomes, and are ultimately transformed into deadly malignant tumors. Two oncogenes, α-HPV E6 and E7 proteins drive this progression by activating telomerase and degrading two tumor suppressor, p53 and pRB (Dyson et al., 1989; Münger et al., 1989; Scheffner et al., 1990; Werness et al., 1990; Huibregtse et al., 1991; Boyer et al., 1996; Klingelhutz et al., 1996; Kiyono et al., 1998; Oh et al., 2001). These oncogenes also induce aberrant activation of the DNA damage response (DDR) and in some case, impair the cells ability to repair DNA damage (Patel et al., 1999; Zimmermann et al., 1999; Moody and Laimins, 2009; Sakakibara et al., 2011; Gillespie et al., 2012; Reinson et al., 2013; Hong et al., 2015; Wallace et al., 2017). In notable contrast to β-HPV associated malignancies, tumors caused by α-HPV are dependent on the continued expression of α-HPV E6 and E7 (Hwang et al., 1993; Goodwin and DiMaio, 2000; Goodwin et al., 2000). Because the β-HPV homologs of these α-HPV oncogenes are believed to be the primary contributors to NMSC, β-HPV E6, and E7 are frequently compared to α-HPV E6 and E7. When informative, we will make similar comparisons.

### Beta-HPV in epidermodysplasia verruciformis patients and organ transplant recipients

The oncogenic potential of β-HPV is most firmly established in people with compromised immune systems. β-HPV associated cSCCs occur in 30–60% of people with the rare genetic disorder epidermodysplasia verruciformis (EV) and presented the first link between β-HPV infections and skin carcinogenesis (Orth, 1986). β-HPV+ tumors found in people with EV generally contain a high copy number (~300 copies/cell) of viral DNA (Dell'Oste et al., 2009). In contrast, β-HPV+ tumors of non-EV patients show a very low viral DNA copy number <1 per cell (Weissenborn et al., 2005; Arron et al., 2011). The most frequently β-HPV types associated with EV are β-HPV 5 and 8 (also less often β-HPV 14 and 20) (de Oliveira et al., 2004; Dell'Oste et al., 2009). Tumors in individuals with EV occur predominantly in parts of the body frequently exposed to the sun, suggesting a role for UV in β-HPV associated tumorigenesis (Pfister, 2003). Another group at risk for β-HPV associated cSCCs are people receiving immunosuppressive therapy following organ transplant. Organ transplant recipients (OTRs) (Bouwes Bavinck et al., 2001) show an increased susceptibility to β-HPV infections, a higher prevalence of viral DNA and a greater risk of developing non-melanoma skin cancer (Boyle et al., 1984; Kiviat, 1999). This increased risk is particularly notable if they are seropositive for β-HPV, with a hazard ratio of 2.8 (Genders et al., 2015). Both, EV-patients and OTRs display a significantly elevated viral load than the immunocompetent population (Dell'Oste et al., 2009; Weissenborn et al., 2012). Further, OTRs that have similar β-HPV viral loads to patients with EV show a 100-fold increase of cSCC incidence (Weissenborn et al., 2012). Investigations in to NMSCs in EV and OTR patient groups provide strong evidence that β-HPV infections have oncogenic potential. Additionally, the OTR patient group shows that β-HPV associated cSCCs are not limited to patients with the rare EV disorder (Howley and Pfister, 2015; Tommasino, 2017). These “special” scenarios provide the proof of context specific β-HPV induced oncogenesis, but defining the breath of β-HPV's contribution to NMSC development is a critical area of research as millions of people are diagnosed with these malignancies each year.

### Beta-HPV in the general population

β-HPVs inhabit the cutaneous epithelium and are found in abundance throughout the population (Casabonne et al., 2009; de Koning et al., 2009; Weissenborn et al., 2012; Farzan et al., 2013). A particularly frequent site of infection is the hair follicles of eyebrows (Weissenborn et al., 2012; Neale et al., 2013; Iannacone et al., 2014). Unlike α-HPV infections, which are usually sexually transmitted and occur later in life, β-HPV infections often occur during early childhood through skin to skin contact (Antonsson et al., 2003; Weissenborn et al., 2009). The persistence of α- and β-HPVs is a further differentiating factor, although the exact mechanisms driving this difference are not fully appreciated. Median β-HPV infection duration is 8.6 months in eyebrow hairs, while infections of the skin are less common but have a median persistence of 11.3 months (Hampras et al., 2014). In contrast α-HPV infections persist longer (18.3 months on average), and upon accidental genome-integration are present for decades (Richardson et al., 2003). Suggesting reinfections, β-HPV infections can persist within a family for several years without manifestation of clinical symptoms (Hsu et al., 2009). While infections first occur in infants, advanced age is a risk-factor for a β-HPV infection (Hazard et al., 2007; Weissenborn et al., 2009). Sunburn is another risk factor, potentially due to local immune suppression (Hampras et al., 2014). The evidence of β-HPV's involvement in cSCCs of EV patients led to the classification of HPV 5 and HPV 8 as possibly carcinogenic by a WHO-IARC 2009 work group (Bouvard et al., 2009).

### Epidemiology

Epidemiologically, β-HPV antibody positivity is associated with an increased risk for cSCCs, especially for infections by β-HPV 38 (Bzhalava et al., 2013; Chahoud et al., 2016). There is also a difference of β-HPV prevalence by anatomical site (Hampras et al., 2017). The most common site of infection was genital skin (81.6%), followed by forearm skin (64.4%), eyebrow hairs (60.9%), oral mucosa (35.6%), and anal mucosa (33.3%). The most common type on the sunlight exposed and therefore risk associated sites eyebrows and forearm are β-HPV 38 and β-HPV 12 respectively. High loads of β-HPV DNA are statistically associated with increased cutaneous SCC incidences at an odds ratio of ~3 in immunocompetent Australians and immunosuppressed OTRs (Bouwes et al., 2010; Neale et al., 2013). People with cSCCs more frequently test positive for viral DNA in skin as well as anti β-HPV L1 antibodies than the general population (Forslund et al., 2007; Waterboer et al., 2008; Karagas et al., 2010; Iannacone et al., 2012; Farzan et al., 2013). The involvement of β-HPV in cSCC carcinogenesis is somewhat challenged by the low relative incidence rate of β-HPV+ cSCCs considering the high prevalence of 80% for β-HPV infections. Furthermore, unlike α-HPV+ cancers, the expression of β-HPV viral proteins is not required for tumor maintenance and the viral DNA copy-number is <1 per cell in β-HPV associated cSCCs (Meyer et al., 2001; Nindl et al., 2006). Since cSCC tumors typically occur in parts of the body exposed mutagenic UV radiation from the sun, the role of β-HPV is thought to be in the initiation and acceleration of genomic destabilization.

The phrase “hit and run” was coined to describe the hypothesize contribution of β-HPV infections to NMSCs, where the virus acts as a cofactor along with UV during tumor initiation (Bavinck et al., 2008). The destabilization of the host genome caused by sun exposure is augmented by β-HPV's ability to attenuate the cellular response to UV damage and thus increase the risk of oncogenic mutations capable of driving tumor development independent of continued β-HPV gene expression. Epidemiological studies support this hypothesis (Forslund et al., 2007; Iannacone et al., 2012) as there is increased prevalence and frequency of β-HPV in precancerous, actinic keratinosis lesions (AK). AK, otherwise known as solar keratinosis, is an abnormal growth of the skin, induced by UV exposure (Moy, 2000). Consistent with a role in tumor initiation, despite being found at very low copy numbers in cSCCs, the β-HPV viral load in AK is >50 copies/cell (Weissenborn et al., 2005). Together these data form the foundation that supports the “hit and run” model of β-HPV associated skin cancer, but this hypothesis is dependent on the ability of β-HPV infections to make UV more mutagenic.

### DNA damage response-pathways

**Figure 1:**
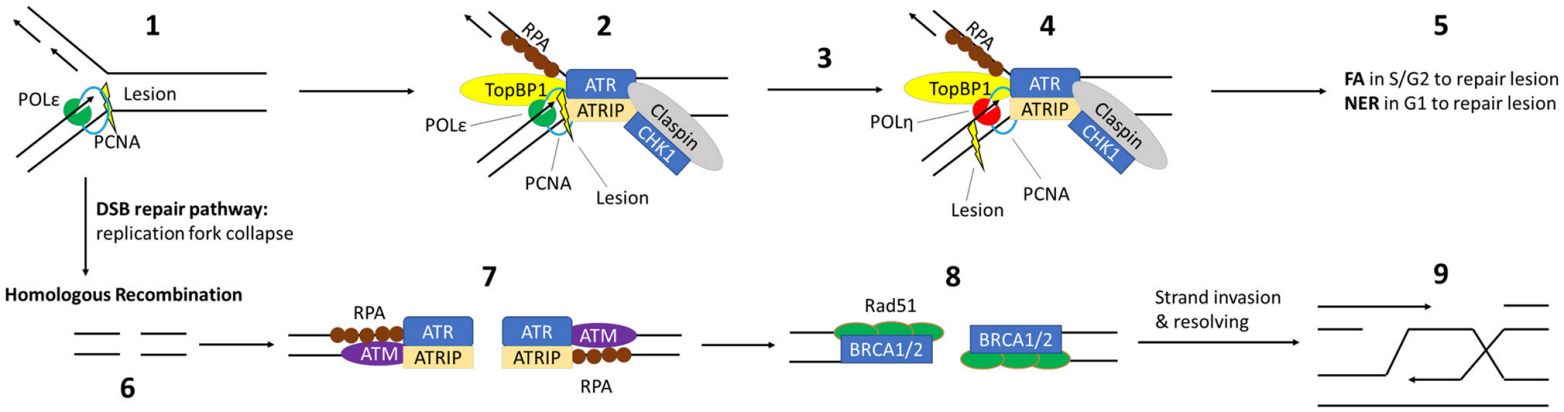
Critical DDR Pathways for UV induced DNA Damage: (1) UV induces an intrastrand lesion, causing the replication fork to stall in S-pase. (2) Exposed ssDNA is coated with RPA, followed by recruitment of TopBP1 and ATR/ATRIP. TopBP1 accelerates autophosphorylation of ATR. Claspin and CHK1 are recruited and phosphorylated, promoting fork stabilization. (3) This leads to Rad6/Rad18 recruitment and mono-ubiquitination of PCNA (not shown), triggering the switch from high fidelity replication DNA-polymerases to y-family polymerase Polη. (4) Then, Polη replicates past the lesion and normal replication can continue after a repeated polymerase switch back to the high-fidelity replication polymerase. (5) The lesion itself is repaired by either the FA or NER pathway. (6) Prolonged stalling leads to replication fork collapse into a DSB. The MRE11, Rad50 and Nbs1 complex (not shown) is recruited to this DSB, initiating strand resection and recruitment/activation of ATM. (7) After strand resection, the exposed ssDNA is coated by RPA and ATR/ATRIP is recruited. (8) RPA is then replaced by RAD51, with the assistance of BRCA1 and BRCA2. (9) This facilitates homology-dependent single strand invasion and resolution of the lesion.

The most likely way that β-HPV could amplify the destabilization of the host genome induced by UV is through the aberration of the cellular DDR. The primary type of DNA damage caused by UV are DNA intrastrand crosslinks, most often cyclobutane purimidine dimers or CPDs (Yang, 2011). Failure to properly repair these lesions can result in point mutations (Brash et al., 1991; Keohavong et al., 1991). Crosslinked DNA can also cause a replication fork collapse and subsequent double strand break in DNA (DSB) (Jeggo and Löbrich, 2007). If DSBs are not repaired, the damage becomes much more deleterious, including chromosome rearrangement or loss of entire chromosome arms (Pankotai and Soutoglou, 2013). In this section, we will introduce the biochemical pathways that coordinate the cellular response to UV-damage to preserve the integrity of the human genome. A graphical representation of the cellular response to UV induced DNA damage in S-phase can be found in Figure 1. During S-phase, translesion synthesis (TLS) prevents UV-induced DNA crosslinks from causing replication fork collapse by facilitating the bypass of the damaged bases (Lerner et al., 2017). Depending on the position in the cell cycle, nucleotide excision repair (NER), and the Fanconi Anemia (FA) pathways repair these crosslinks (Moldovan and D'Andrea, 2009; Marteijn et al., 2014). While FA requires sister chromosomes to complete repair and is thus limited to cells that have undergone replication, NER is not bound by cell cycle position. UV-induced DSBs are the result of replication fork collapse and therefore must occur during S-phase. Although DSB repair occurs through two major pathways, non-homologous end joining (NHEJ) and homologous recombination (HR), DSBs occurring during S-phase are repaired predominantly by HR (Mao et al., 2008). Repairing DNA lesions usually requires pausing the cell cycle to avoid escalating the damage during replication (Willis and Rhind, 2009). Finally, should the cell receive more damage than can be repaired, the intrinsic apoptosis pathway will initiate program cell death (Offer et al., 2002).

Although recognized as distinct pathways, the individual proteins that make up these repair mechanisms are often shared. This is particularly true for repair kinases (Yang et al., 2003; Yan et al., 2014). For instance, ATR controls the rate limiting step of NER (through phosphorylation mediated stabilization of XPA) and facilitates translesion synthesis by phosphorylating the TLS-specific polymerase, Polη (Chen et al., 2008; Göhler et al., 2011; Lee et al., 2014). ATR's kinase activity is similarly necessary for the repair of crosslinked DNA by the FA pathway and plays a role in DSB repair via the homologous recombination pathway (Jazayeri et al., 2006; Shiotani and Zou, 2009; Shigechi et al., 2012; Maréchal and Zou, 2013). A similar situation exists for the related kinase, ATM. ATM phosphorylates proteins critical for the FA, NER, and HR repair pathways (Ray et al., 2013, 2016; Shiloh and Ziv, 2013). It may also play a role in translesion synthesis. Further, both ATM and ATR can stabilize p53 in response to DNA damage, causing p53-dependent DNA repair or apoptosis (Kruse and Gu, 2009; Cheng and Chen, 2010). ATM and ATR are both classically involved in DNA damage induced cell cycle arrest (Reinhardt and Yaffe, 2009). Both of these kinases also help pause the cell cycle progression by phosphorylating/activating cell cycle regulatory proteins (CHK1 and CHK2) (Branzei and Foiani, 2008). Finally, ATM and ATR also facilitate programed cell death or apoptosis should the cell's DNA be too extensively damaged. Specifically, their phosphorylation of p53 stabilizes the tumor suppressor and can result in p53-dependent apoptosis (Banin et al., 1998; Tibbetts et al., 1999; Shiloh and Ziv, 2013).

The RPA complex (RPA14, RPA32, and RPA70), BRCA1 and BRCA2 are similarly important for a myriad of DDRs (Yoshida and Miki, 2004; Maréchal and Zou, 2015). The RPA heterotrimer binds single stranded DNA (ssDNA) protecting it from degradation. Because ssDNA intermediates occur during both homologous recombination and translesion synthesis, RPA proteins are essential for these repair mechanisms. Both the FA and HR pathways require BRCA1 and BRCA2. FA pathway proteins contain the prefix “FANC” in their names indicating that many of these proteins were named after loss of the gene was shown to result in the clinical manifestation (Fanconi Anemia) from which the repair pathway derives its name, for example, FANCA, FANCB, FANC. Indicative of its requirement for FA repair an alternative name for BRCA2 is FANCD1. Moreover, BRCA1 was recently shown to be a critical component of the FA pathway (Domchek et al., 2013; Sawyer et al., 2015) and even described as the pathway's “missing link” (D'Andrea, 2013). The requirement of BRCA1 and BRCA2 in repair of DSBs by homologous recombination has been established for over a decade. Further indicative of how intertwined the cellular DNA response is, ATR and ATM both play regulatory roles in repair by phosphorylating the RPA complex, BRCA1 as well as BRCA2.

## Effects of β-HPV E6 on DNA damage response

### Inhibition of apoptosis

The E6 protein of β-HPV can hinder both DNA repair machinery and apoptotic pathways in response to DNA damage. Unlike high risk α-HPVs, it does not commonly degrade the ”guardian of the genome,” p53, one of the most prominent high-risk E6 targets, directly (Scheffner et al., 1990; White et al., 2014). The tumor-suppressor, p53, is an important signaling protein that regulates two large subsets of target proteins: negative regulators of cell cycle progression (p21, 14-3-3, GADD45α) and pro-apoptotic proteins (PUMA, BAX, BAK). In normal cells, p53 has to balance on a fine line between sufficient activity to ensure genome fidelity and tumor suppression as well as avoiding hyperactivity that would induce abnormal aging by depleting stem-cell populations (Vogelstein et al., 2000; Reinhardt and Schumacher, 2012). Compared to high risk α-HPV E6s, β-HPV E6, apart from β-HPV 49, have a lower affinity for a cellular E3 ubiquitin ligase, known as E6AP or UBE3A, and are therefore not able to form the E6-E6AP complex required for proteasomal degradation of p53 (Huibregtse et al., 1991; Cornet et al., 2012). Instead, β-HPV types 17, 38, and 92 have been shown to bind to p53 directly and stabilize it (White et al., 2012), while β-HPV 23 has been shown to interfere with the phosphorylation-dependent activation of p53 by inhibiting HIPK2 (Muschik et al., 2011). β-HPV 5 and β-HPV 8 delay the stabilization and phosphorylation after UV irradiation (Wallace et al., 2014). HIPK2 is a protein kinase that can phosphorylate p53 at Ser 46, which subsequently leads to the acetylation of p53 at Lys 382 and a promotion of p53 target gene expression. More specifically, HIPK2's activity is UV-induced and UV exposure leads to HIPK2 mediated growth arrest or apoptosis via p53. β-HPV 38 E6 induces the accumulation of ΔNp73, altering p53 functions (Accardi et al., 2006). These effects cause the alteration or loss of p53's activity as a transcriptional co-factor and impact both apoptosis- (Fas, BAX) and cell cycle checkpoint pathway (p21) gene expression (White et al., 2014). p53 is also a known transcription factor for the TLS polymerase Polη, implying the TLS DDR pathway as a suitable target for investigation (Lerner et al., 2017).

**Figure 2:**
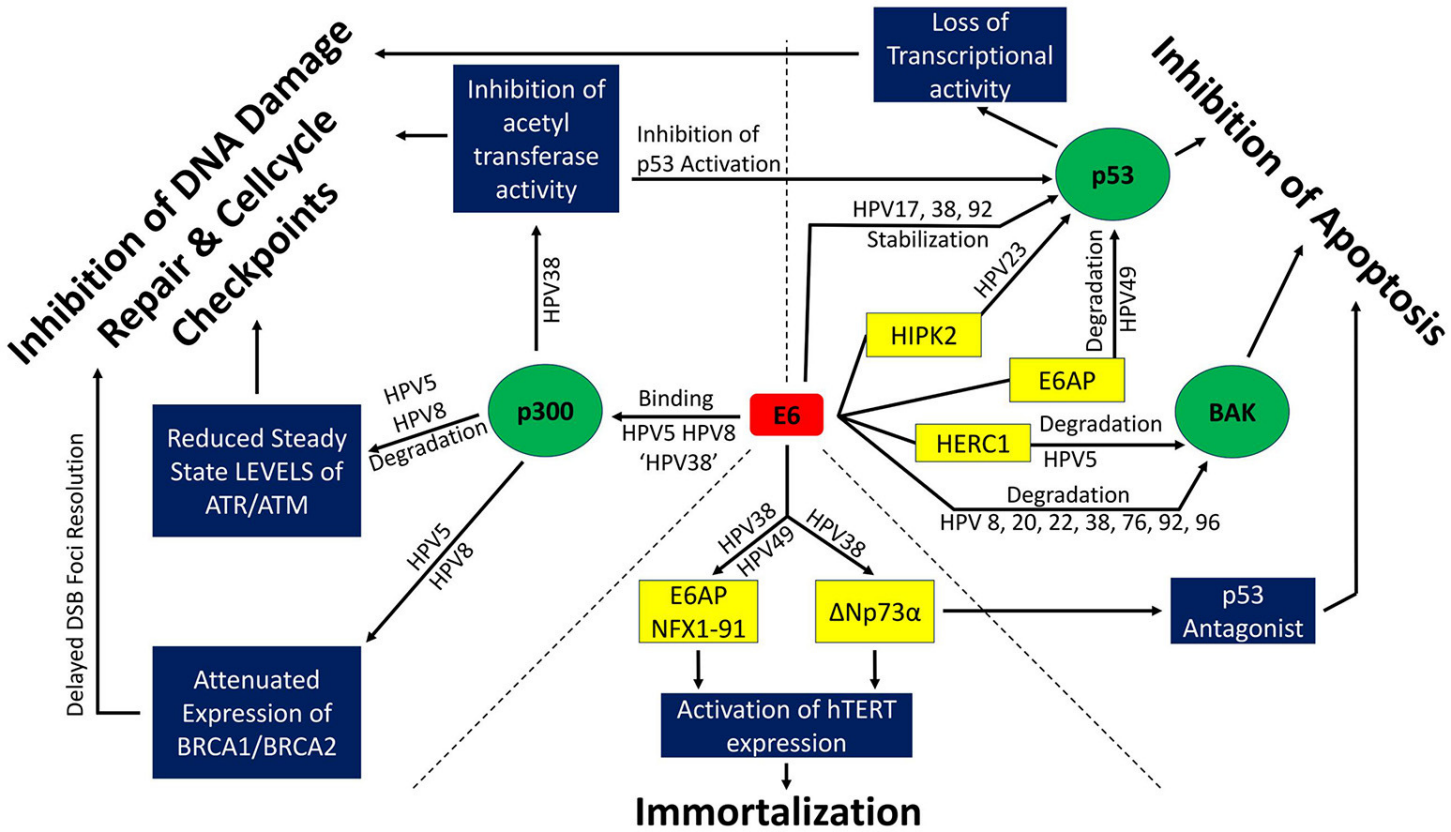
β-HPV E6 and its effects on genome stability. Interactions among β-HPV E6s and proteins involved in DNA repair and cell cycle check points (upper left third), apoptotic signaling (upper right third), and immortalization (bottom third) are depicted. Green circles denote cellular proteins that are central to β-HPV E6's ability to alter the cellular response to DNA damage. Blue boxes depict the diversity of cellular processes altered through the manipulation of the green circular proteins. Yellow boxes represent proteins that cooperate in the manipulation of the green circular proteins or facilitate immortalization. ‘HPV 38’ refers to the fact that while β-HPV 38 E6 can bind p300, it does not bind it strong enough to destabilize the histone acetyltransferase.

The selection between a p53-mediated checkpoint activation and p53-mediated apoptosis depends on a cell type specific threshold of p53 expression and activation (Kracikova et al., 2013). Mild DNA damage triggers p53-dependent checkpoint activation and subsequent DNA repair while moderate DNA damage causes p53-mediated senescence. Excessive DNA damage or failed cytokinesis induce apoptosis (Chen et al., 1996). β-HPV E6 can also inhibit apoptosis downstream of p53. The interaction of E6 with a pro-apoptotic protein, Bcl2 homologous antagonist killer (BAK), is a highly conserved function across HPV types and genera (Thomas and Banks, 1999; Simmonds and Storey, 2008; Underbrink et al., 2008; Jackson and Bartek, 2009; Holloway et al., 2015; Tomaić, 2016). Increases in BAK abundance are an essential step in the intrinsic apoptotic pathway (Chittenden et al., 1995). β-HPV E6 prevents the accumulation of Bak following UV-irradiation inducing DNA damage. An overview of the different pathways for β-HPV E6 mediated inhibition of apoptosis and the β-HPV types involved can be found in the upper right panel of Figure 2.

### Interference with checkpoint signaling & DNA damage repair

Upstream of the inhibition of apoptosis, β-HPV E6 can attenuate G1 to S-phase cell cycle checkpoint induction in response to DNA damage (Wallace et al., 2012; Hufbauer et al., 2015) and continue cellular proliferation while DNA damage repair is attenuated (Giampieri and Storey, 2004). These effects arise from the interaction of β-HPV E6 with the acetyltransferase p300 (Muench et al., 2010; Howie et al., 2011). As an important coactivator of DDR gene transcription, the loss of p300 activity has far reaching consequences. β-HPV 5 and 8 have been shown to facilitate the destabilization of p300 (Howie et al., 2011; Wallace et al., 2012, 2013), while β-HPV 38 has been shown to inhibit p300 acetyltransferase activity (Muench et al., 2010). The steady state levels of the DDR kinases ATM and ATR as well as BRCA1 and BRCA2 are reduced because of the lack of p300 in β-HPV 5 and β-HPV 8 E6 expressing cells (Wallace et al., 2012, 2013, 2015). This attenuates the repair of UV induced cyclobutane pyrimidine dimer (CPD), increases the UV-induced frequency of DSBs and attenuates LINE-1 retrotransposition. Double strand breaks can be a secondary effect of UV-induced DNA damage, occurring when a replication fork collapses at an unrepaired CPD. Both the repair of CPDs (ATR dependent) as well as the repair of DSBs (ATM, BRCA1 and BRCA2 dependent) are significantly delayed by β-HPV 5 and β-HPV 8 E6 (Giampieri and Storey, 2004; Wallace et al., 2012, 2015). γH2AX is a known marker for DSBs and its foci kinetics can be utilized as indicators for DSB-repair. The (β-HPV 5 E6 and 8 E6) p300 degradation-dependent attenuation of BRCA1/BRCA2 expression and foci formation led to delayed γH2AX foci resolution after DSBs were induced by ionizing radiation (Wallace et al., 2015).

ATR and ATM activate cell cycle checkpoints by phosphorylating checkpoint kinases Chk1 and Chk2 respectively. Activation of Chk1/Chk2 induces cell cycle arrest and DDR (Reinhardt and Yaffe, 2009). β-HPV 5 and 8 E6 reduce steady state levels of ATM and ATR *in vitro* as well as reduced pATM and pATR levels *in vitro* and *in vivo* (Hufbauer et al., 2015). This leads to an attenuated Chk1 phosphorylation and impaired G1 to S-phase checkpoint activation, presenting a bypass to an important tumor-suppressing barrier. Furthermore, p300 is an important transcriptional cofactor for BRCA1, BRCA2 (Both essential proteins for HR) (Goodman and Smolik, 2000; Pao et al., 2000; Yoshida and Miki, 2004) and has been shown to be essential for proper cytokinesis and the faithful resolution of mitotic figures (Turnell et al., 2005; Xia et al., 2012). Attenuation of proper checkpoint signaling and the consequent possible unscheduled cell cycle progression leaves pre-existing DNA damage unaddressed. This allows less severe types of damage (for example, UV-induced intrastrand crosslinks) to evolve into more deleterious types of DNA damage like DSBs. β-HPV E6 expressing cells have been shown to contain a significantly larger portion of multinucleated cells *in vitro* (Wallace et al., 2014), pointing to a possible attenuation of the Hippo pathway. The Hippo pathway stabilizes p53, inactivates the oncogene YAP and induces apoptosis in the event of failed cytokinesis (Harvey et al., 2013; Ganem et al., 2014). The mechanisms through which β-HPV E6 disrupts the DDR are depicted in the upper left panel of Figure 2.

In addition to p300-dependent checkpoint inhibition, β-HPV 5E6 and 8E6 can bind the transcription factor SMAD3, thereby inhibiting the transforming growth factor-beta (TGF-β) pathway (Mendoza et al., 2006). TGF-β has a well-described dual role in carcinogenesis. In normal cells and early carcinomas, it acts as tumor suppressor through cytostatic effects. As the cancer progresses, the TGF-β aids proliferation, survival, invasion, and angiogenesis of tumor cells (Lebrun, 2012). Destabilization of the SMAD3 may attenuate these tumor suppressive effects by inhibiting the expression of CDK inhibitors p16, p17, p21, and p27 and possibly promoting unscheduled cell cycle progression from G1- into S-phase (Itoh et al., 2000; ten Dijke and Hill, 2004). Disruption of the TGF-β pathway has also been linked to an impaired function of DSB-repair following ionizing radiation (Kirshner et al., 2006; Bouquet et al., 2011; Kim et al., 2015).

### Additional functions of β-HPV E6

Additional notable functions of β-HPV E6 include hTERT stabilization (β-HPV 5, 20, 22, 38; Bedard et al., 2008; Gabet et al., 2008; Cornet et al., 2012), which may stabilize the genome by preventing unstably short telomers. Conversely, it could decrease genome fidelity by aborting the role of telomers as a “cellular clock” that prevent proliferation of older cells that are more likely to contain mutations (Sharma et al., 2003). Another target of β-HPV 5 and 8 E6 is the Notch signaling pathway. The Notch signaling pathway has been shown to have tumor suppressive functions in epithelial cells (Reichrath and Reichrath, 2012) and can decide cell fate (Lai, 2004). It can also act as a negative regulator of the ATM-dependent DDR (Vermezovic et al., 2015). β-HPV 5 and 8 interfere with the Notch signaling pathway through interaction with MAML1. The β-HPV E6-mediated repression of Notch signaling delays S-phase cell cycle exit and differentiation (Brimer et al., 2012; Meyers et al., 2013). Prolonged or perpetual existence in S-phase in combination with impaired DNA-damage signaling and attenuated DDR could lead to cross-amplification of these individual effects.

### Summary

β-HPV E6 attenuates UV-induced apoptosis and DDR through interaction with multiple targets. BAK degradation and prevention of apoptosis is highly conserved throughout the β-HPV genus. Less common is the inhibition of apoptosis through direct interaction with and degradation of p53 (β-HPV 49, E6AP mediated) or the inhibition of p53 activation (β-HPV 23 through HIPK2 interaction). β-HPV 17, 38, and 92 E6 interact with p53 directly, leading to its stabilization but altering or restricting p53's transcriptional activity. β-HPV 5, 8, and 38 E6 interfere with DDR and checkpoint signaling through interaction with p300. A combination of inhibited apoptosis, impaired DNA damage signaling and attenuated DSB response bears a potential to destabilize the genome over time or cause carcinogenesis in combination with DNA-damaging agents that is consistent with β-HPV's proposed contribution to skin cancer. Notably, although through markedly different mechanisms, high risk α-HPV oncogenes similarly contribute to tumorigenesis by impairing the cellular response to genome destabilizing events.

## E7 and its influence on cell cycle progression and DDR

### Promotion of cell cycle progression

A canonical function of the high risk α-HPV E7 oncogene is the degradation of the tumor suppressor pRb (Moody and Laimins, 2010). In normal cells, the function of pRb is the suppression of E2F transcription factor dependent genes through binding E2F and inhibiting its association with promoters (Dyson, 1998). E2F-family proteins facilitate cell cycle progression and proliferation by promoting the expression of a variety of genes encoding for proteins involved in G1/S-phase transition (Bertoli et al., 2013). High risk α-HPV E7 oncogenes bind directly to pRb via the highly conserved LXCXE binding motif (Münger et al., 1989). This leads to the disruption of the E2F—pRb complex, allows E2F to bind to its transcriptional targets (Chellappan et al., 1992). E2F target expression (for example cyclin A or cyclin E) then facilitate premature cell cycle progression and S-phase entry (Zerfass et al., 1995). Cell cycle checkpoints are meant to pause the cell cycle in case of DNA damage and allow time to repair the damage, or, if the damage surpasses a threshold, induce senescence or apoptosis. Cell cycle checkpoints are an integral part of a cells quest to maintain genome integrity. Consequently, attenuated checkpoint activation leads to a destabilization of the genome through accumulating DNA damage (Malumbres and Barbacid, 2009). α-HPV E7 allows keratinocytes with damaged DNA to progress through cell cycle check points, even when p53 is induced (Demers et al., 1994). Much like these α-HPV E7s, several of the β-HPV E7s (β-HPV 5, 8, 38, 49) can interact with Rb (Yamashita et al., 1993; Caldeira et al., 2003; Cornet et al., 2012). However, the β-HPV E7 interactions tend to cause a hyperphosphorylation of Rb in Keratinocytes, rather than its destabilization (Caldeira et al., 2003; Cornet et al., 2012). Hyperphosphorylation of Rb inhibits its ability to bind and inactivate E2F, allowing E2F to promote transcription of its target genes (Bertoli et al., 2013). Additionally, β-HPV 24, 38, and 49 E7 severely attenuate pRb half-life in rodent fibroblasts, while β-HPV 14, 22, 23, and 36 do not affect pRb's half-life (Cornet et al., 2012). Cornet et al. also found that in the context of E6 and E7 expressing cells, β-HPV 14 and 22 can degrade pRb, but do not induce the expression of cyclin A or Cdk1. Cdk1 is essential for cell cycle progression(Santamaria et al., 2007).

### Alteration of the p53-transcriptional network

The tumor suppressor p53 is an essential factor in the avoidance of inappropriate cell proliferation in the presence of genotoxic stress. It has transcriptional targets in several pathways, including DNA damage tolerance and apoptosis (Espinosa et al., 2003; Yu and Zhang, 2005; Beckerman and Prives, 2010). While the mechanisms of how high Risk α-HPV E7 disrupts p53 activity remain a poorly defined, significantly more is known about how some β-HPV E7 proteins disrupt this essential tumor suppressor. ΔNp73 is an important antagonist of the p53 gene family and participating in a negative feedback loop with p53 (Bailey et al., 2011). β-HPV-38 E7 promotes the accumulation and stability of ΔNp73α through both transcriptional and post-translational mechanisms (Accardi et al., 2006, p.73; Saidj et al., 2013). β-HPV 38 E7 promotes the accumulation of double monophosphorylated p53 (serine 15 and 392) in the nucleus. These dual p53 phosphorylation events increase ΔNp73α expression. β-HPV 38 E7 also mediates the nuclear translocation of the IκB kinase (IKKβ) that increases the stability of ΔNp73α by phosphorylating it at serine 422 (Accardi et al., 2011). Furthermore, IKKβ, ΔNp73α, DNMT1, and EZH2 form a transcription regulatory complex. This complex is able to bind to a subset of p53 regulated promoters and interferes with the expression of DDR pathway- and apoptosis-related genes, but not with pro-survival genes like Survivin. This implies a role of β-HPV-38 E7 in the inhibition of p53 dependent apoptosis and allows for speculation on the influence of β-HPV E7 over DDR-proteins that depend on p53 activity as a transcriptional factor (for example Polη of the TLS pathway and XPC of the NER pathway) (Fischer, 2017).

### Other functions related to DDR

A systematic screening of E7 interacting protein has shown that E7 from β-HPVs 8, 25, and 92 can interact with the tyrosine phosphatase and tumor suppressor PTPN14. While several high- and low-risk α-HPV types have been shown to degrade PTPN14 (White et al., 2016; Szalmás et al., 2017), the role of the β-HPV E7—PTPN14 interaction is yet to be investigated. PTPN14 is a required regulator of the Hippo signaling pathway, as it is necessary for the translocation of the Hippo transcription factor, YAP1, from the nucleus to the cytoplasm (Wang et al., 2012). This indicates that β-HPV E7 may attenuate the Hippo pathway, if β-HPV E7 proves to also be capable of PTPN14 destabilization. The Hippo pathway plays a crucial role in the control of proliferation and induces apoptosis in the event of cytokinesis (Ganem et al., 2014). Phosphorylation of Yap1, a part of the hippo pathway, is a critical step in the induction of apoptosis in response to DNA damage (Levy et al., 2008). β-HPV infections occur in the skin and thus in an environment where UV-induced cell cycle arrest and apoptosis occur with some frequency. As a result, it would not be surprising if there yet undiscovered abilities of β-HPV E7 to disconnect the cellular response to UV in manners that promote the β-HPV lifecycle.

## Mouse models: evidence for a role of β-HPV in carcinogenesis via a hit and run mechanism

Mouse models can be an effective tool for elucidating the oncogenic potential of β-HPVs. The most common of these is the transgenic (tg) mouse model that expresses can individual and groups of β-HPV genes (E2, E6, E7, E6/E7) or the entire early region of the virus under a keratin promoter. Placing the expression under a keratin promoter restricts expression to keratinocytes, the cell type infected by β-HPV. The result is that genes are only expressed at anatomical sites relevant in the context of β-HPV infections. Our review of the literature shows that β-HPV 8 is the most frequently investigated β-HPV type, followed by β-HPV 38, β-HPV 49, and β-HPV 20. Additionally, one study has investigated β-HPV 5 E7 in a nude mouse/artificial skin graft model (Buitrago-Pérez et al., 2012).

### β-HPV 8 transgenic-mouse models

Schaper et al. established the first β-HPV tg-model using the Keratin 14 (K14) promoter to express the entire early gene region of β-HPV 8. Transgene expression was the highest for E2, followed by E6 and E7 (Schaper et al., 2005). While the tg-negative littermates did not develop lesions on the skin or other organs, 91% of the β-HPV 8 tg-positive mice developed single- or multifocal benign tumors. Of this population, 25% showed varying degrees of dysplasia and finally 6% developed SCCs spontaneously. The SCCs developed without additional DNA-damaging events. This established an *in vivo* link between β-HPV 8 and carcinogenesis. A caveat, however, is the stable oncogene expression in tg-mice. β-HPV infections, and therefore expression of β-HPV genes in the immunocompetent population, are transient.

In a follow up study, a possible role of β-HPV 8 E2 in skin tumor induction was revealed when 60% of the E2 positive population, but none of the E2 negative population developed spontaneous ulcerous lesions of the skin (Pfefferle et al., 2008). Moreover, 3 weeks after UV irradiation, 87% of a tg-mouse line with high E2 expression levels and 36% of a tg-mouse line with lower E2 expression levels developed skin tumors. On the other hand, irradiation of tg-negative mice did not lead to tumor formation. This could point toward a potential synergy between β-HPV 8 E2, E6, and E7 for their role in carcinogenesis.

Later, by expressing E6 individually under the K14 promoter, it was shown that β-HPV 8 E6 is the major driving force behind both spontaneous tumor development in tg-mice and that tumor formation could be prevented via DNA vaccination (Marcuzzi et al., 2009, 2014). Rapid tumor development by induction, either through UV irradiation or wounding, was demonstrated. This observation that persistent β-HPV 8 infections combined with UV exposure and wound healing processes pose a significant risk factor for skin cancer is consistent with observations in individuals with persistent β-HPV 8 infections due to EV. A potential role of the signal transducer and activator of transcription 3 (Stat3) was demonstrated in tg mice expressing the early region of β-HPV 8 that additionally had an epidermis restricted ablation of Stat3 (Andrea et al., 2010, p.3). The Stat3 heterozygous line was significantly less prone to spontaneous tumor development and these tumors did not progress to malignancy. Hufbauer et al. showed that β-HPV 8 E6 expressing tg-mice dysregulate mi-RNA expression (Hufbauer et al., 2011). Following UV induced inflammation and wound healing, the levels of mi-RNA for regulatory targets including cell-cycle (Rb) and apoptosis (PTEN, PDCD4) remained dysregulated, while dysregulation in wt-mice was transient. Hufbauer et al. also demonstrated that β-HPV 8 E6 expression leads to an impaired DNA-damage response in tg-mice following UV irradiation (Hufbauer et al., 2015). β-HPV 8 E6 expressing mice were shown to be thymine-dimer positive for an extended period while wt-mice efficiently repaired the UV induced DNA damage within 48 h. More importantly, probing for the DSB marker, γH2AX, revealed that the persistence of UV induced lesions in β-HPV 8 E6 expressing cells ultimately led to the formation of highly mutagenic and more dangerous DSBs. Immunohistochemical analysis of tg-mouse skin biopsies showed an increased signal for γH2AX at both, early (3d, 5d) and later (13d, 24d) timepoints. This not only demonstrates β-HPV 8 E6's attenuation of DDR, but also its ability to enhance the cells tolerance of persistent and increasingly severe DNA-damage. UV radiation typically causes intrastrand lesions that are tolerated by TLS- and repaired by NER-pathway. If this type of damage persists it will lead to increased rates of DSBs. The emergence and persistence of DSBs could point toward a broad influence of β-HPV 8 E6 on the DNA-damage response machinery, stretching from pathways meant to handle minor DNA damage (TLS, NER) to pathways that control and repair highly mutagenic DSBs (NHEJ, HR).

The role of β-HPV 8 E7 has also been investigated in tg-mice (Sperling et al., 2012; Heuser et al., 2016). It was shown that β-HPV 8 E7, but not β-HPV 8 E6 enables an escape from the immune response of β-HPV 8^+^ lesions. β-HPV 8 E7 achieved this by inhibiting chemotactic signaling. β-HPV 8 E7 binds to C/EBPβ and inhibits its interaction with the CCL20 promoter, leading to decreased levels of CCL20, a chemoattractant for Langerhans cells. β-HPV 8 E7 is also critical for the invasiveness of hyperproliferating keratinocytes by dysregulating cell-cell interactions.

### β-HPV 38 transgenic-mouse models

While β-HPV 8 tg-mice demonstrated spontaneous tumor formation, tg-models of other β-HPV types do not exhibit the same behavior. Dong et al. showed that a mouse model for β-HPV 38 E6/E7 expressing mice under the Keratin 10 promoter exhibit hyperproliferation of the skin, but require external stimuli for carcinogenesis (Dong et al., 2005). Furthermore, ΔNp73α has been identified as integral to the attenuation of cell cycle arrest after UV exposure (Dong et al., 2007). Loss of p53 attenuated ΔNp73α expression and partially restored cell cycle arrest while loss of p73 lead to loss of ΔNp73α and consequently to high levels of p21 expression and cell cycle arrest after UV exposure. Differential susceptibility to chemically induced (DMBA/TPA) carcinogenesis correlates with differential oncogene expression levels and indicates a dose-dependency for risk of carcinogenesis. Irradiation of wt-mice with a single UV-dose led to accumulation of p21 and cell cycle arrest in the epidermis, while β-HPV 38 E6/E7 expressing mice inhibited cell cycle arrest through attenuation of p21 accumulation. Chronic irradiation of tg-mice with UV led to the formation of AK-like lesions that are considered precursors to SCCs in humans, while wt-mice did not develop lesions. Some of the AK-like lesions progressed to SCCs after 22 weeks (Viarisio et al., 2011). This provides evidence for the carcinogenesis-risk amplifying potential of the β-HPV 38 oncogenes E6 and E7.

### Other β-HPV transgenic-mouse models

The K14 tg-model for β-HPV 49 E6/E7 does neither show spontaneous tumor development nor tumor development after UV-irradiation (Viarisio et al., 2016). However, the β-HPV 49 tg-mice lines where susceptible to chemically induced carcinogenesis of the upper digestive tract. When exposed to 4 nitroquinoline 1-oxide (4NQO), 87% of the β-HPV 49 tg-mice developed tumors in the upper digestive tract while 4NQO treatment had little effect on β-HPV 38 tg-mice, which are susceptible to UV-triggered skin carcinogenesis.

A model for β-HPV 20 E6/E7 showed enhanced proliferation and papilloma formation in the evaluated transgenic lines, when compared to non-transgenic controls (Michel et al., 2006). Chronic exposure to UV radiation led to the development of SCCs and proliferation was enhanced for several weeks after UV treatment. The tg-lines also showed a reduced expression of differentiation markers (involucrin & loricrin and irregular patterns of p53 expression post UV, while controls showed a continued expression of proliferation markers and even expression of p53.

### Summary

Mouse models provide a valuable *in vivo* tool to dissect the relative ability of β-HPV genes to induce tumorigenesis. They also allow a distinction between different carcinogenic potentials exhibited by E2, E6, or E7 to be accessed. The mouse models make it evident that β-HPV E6 carries a particularly high potential due to its effects on both apoptotic pathways and the DDR. The tg-mouse models also support the “hit and run” hypothesis regarding β-HPV induced malignancies, since most models require external stimuli to induce malignant tumors. β-HPV 8 has the potential for spontaneous transformation in tg-models, but it remains unclear whether the potential persists when β-HPV 8 early gene expression is transient, as it is the case in most humans. It also should be pointed out that the average daily UV dose of an American is ~94 J/m^2^ (Godar et al., 2001), while the dosages used in the β-HPV tg-model studies discussed here reach 45-fold great levels. Although constrains in experimental design may require doses of this magnitude, it is a necessary caveat to considered when evaluating tg models of β-HPV induced skin cancer.

## Conclusions

The impact of genus β-HPV on the DNA damage as well as the ability of these viruses to induce carcinogenesis is diverse and manifest in both *in vitro* and *in vivo* investigations. Generally, β-HPV viruses effect on DDR may be explained by their interest of uninterrupted proliferation, despite the presence of DNA damage. While most β-HPV E6 inhibit apoptosis by degrading the pro-apoptotic protein BAK, only a subset (β-HPV 5 and 8 E6) bind p300 strongly enough to destabilize the histone acetyltransferase, causing delayed repair of thymine dimers and double strand breaks (Giampieri and Storey, 2004; Muench et al., 2010; Howie et al., 2011; Hufbauer et al., 2011; Wallace et al., 2012, 2013, 2015). A larger subset of β-HPV E6 proteins (β-HPV 5, 8, 17, 23, 38, 49, and 92) interfere with p53 activity, but they do so through different mechanisms. Continuing the theme of diversity among β-HPVs, β-HPV 38 E6 immortalizes cells through a ΔNp73α-dependent mechanism, while β-HPV 49 E6 acts more similarly to high risk α-HPV E6, immortalizing cells through interactions with E6AP and NFX1-91 (Caldeira et al., 2003; Gabet et al., 2008; Muench et al., 2010). Figure 2 depicts these diverse effects of β-HPV E6.

There are notable differences between β-HPV E7 proteins as well. A large cohort of these viral proteins (β-HPV 5, 8, 24, 38, and 49 E7) impair pRB function. The sole mechanism of pRB interference by β-HPV 5 and 8 E7 is through binding and induction of hyperphosphorylation of pRB. In addition to promoting the phosphorylation of pRB, β-HPV 38 and 49 E7 also decrease the half-life of pRB. β-HPV 24 E7 is only known to reduce pRB's half-life. Further, β-HPV 38 E7 has the ability to interfere with p53 activity by stabilizing the p53 antagonist, ΔNp73α, and increase inhibitory post-translational modifications of p53.

Not surprisingly, *in vivo* transgenic mouse models of β-HPV associated tumorigenesis reflect the varied ability of β-HPV to degrade the fidelity of host cells (Dong et al., 2005; Michel et al., 2006; Viarisio et al., 2011). The tg-mouse models of β-HPV 8 show spontaneous tumor formation (Schaper et al., 2005; Pfefferle et al., 2008; Marcuzzi et al., 2009). While carcinogenesis is augmented by UV exposure in β-HPV 8 mouse models, similar models of other β-HPV induced cancers (β-HPV 20, 38, and 49) require external stimulants to develop tumors. The required stimulants vary as well. Expression of β-HPV 20 and 38 proteins cause cancer in mice exposed to UV, but only β-HPV 49 proteins cause upper digestive tract tumors after exposure to 4NQO. Notably, the transient nature of β-HPV infections in immunocompetent individuals is not reflected in the mouse models discussed above and the ability of persistent β-HPV infection to cause cancer is generally accepted. Further, the amount of UV exposure necessary to cause tumors in β-HPV tg mice is often well above what humans typically receive. Thus, new *in vivo* models will be quintessential to drive the field forward and may include mice infected with the murine papillomavirus (MmuPV1) that recapitulates some but not all of the characteristics of β-HPV (Meyers et al., 2017).

The bulk of evidence for β-HPV infections playing a role in NMSCs suggests that β-HPV 5, 8, and 38 are the most tumorigenic, with a particular and unsurprising emphasis on the homologous of high risk α-HPV oncogenes (E6 and E7). These viruses are expected to promote carcinogenesis via the “hit and run” model that is dependent on their amplification of UV's genome destabilizing effects and otherwise decreasing genome fidelity. Since the mechanisms of abrogating genome stability vary among β-HPVs, it is reasonable to expect that the carcinogenic potential of individual β-HPVs depends on their cellular environment during infection. As a result, not only is the clear delineation of pathways and proteins impacted by β-HPV necessary to understand their oncogenic potential, but also the relative ability among β-HPVs to disrupt these pathways. A major knowledge gap regarding β-HPV induced oncogenesis is the extent that the reduced availability of ATM, ATR, BRCA1, and BRCA2 affects the activity of these proteins in each of the repair pathways that they participate in. This could expand the known ability of β-HPV proteins to disrupt DNA repair to include nearly every cellular response to damaged DNA. Alternatively, if the impairment of these repair proteins was context specific, it could highlight particularly deleterious situations for a β-HPV infection to occur. Further, there is a diversity of genome destabilizing events beyond DNA damage (failed cytokinesis, centrosome duplication errors, etc.) that if disrupted would fit the ascribed “hit and run” model of tumorigenesis. Defining the mutagenic potential of β-HPV infections is essential to take next steps in preventing disease associated with these viruses. Table 1 compares β-HPV 5, 8, 38, and 49 as well as high-risk α-HPV oncogenes regarding their oncogenic potential. A possible intervention is the development of β-HPV specific vaccines. While β-HPV infection occurs in early childhood, a vaccine against specific “high-risk” types may prevent re-infection. Additionally, immunity acquired through vaccination may be more effective than immunity through infection. The FDA-approved technology to make the current vaccine could be readily adapted to prevent β-HPV infections or specific inhibitors of β-HPVs could be developed and added to sunscreens to precisely target the intersection of UV and viral infection. A commonly stated challenge to the development of a vaccine against β-HPV is the fact that these infections are initially acquired soon after birth and that β-HPV infections do not illicit a protective immune response. Although true, vaccination could mitigate disease by preventing reinfection and potential initial infection with particularly oncogenic β-HPVs not necessarily acquired at birth. Further, vaccination is effective against members of the alpha HPV genus despite poor natural adaptive immune responses to those infections. Ultimately, these ambitious goals are dependent on advancements in the dissection of β-HPV biology and its ability to hinder the cellular DDR.

**Table 1:** Characteristics and differences of several β-HPV strands and high risk α-HPVs.

	**HPV5**	**HPV8**	**HPV38**	**HPV49**	**high risk αHPVs**
Impact on DDR	Delay UV repair Destabilize p300 ATM, ATR, BRCA1/2 ↓	Delay UV repair Destabilize p300 ATM, ATR, BRCA1/2 ↓	Delayed UV repair ΔNp73α ↑ Inhibit p300	Unknown	Attenuate and repurpose several DDR proteins
Prevention of Apoptosis	Degrade BAK	Degrade BAK	Stabilize p53 Alter p53 activity Degrade BAK	Degrade p53	Degrade p53
Proliferation	Hyperphosphorylate pRb	Hyperphosphorylate pRb	Attenuate p21 accumulation Phosphorylate pRB	Hyperphosphorylate pRb	Degrade pRb
Immortalization	No	No	Yes	Yes	Yes
Tumor maintenance	Not required	Not required	Not required	Not required	Required
E6 motif	No PDZ domain	No PDZ domain	No PDZ domain	No PDZ domain	PDZ domain present
Animal Models	Artificial skin graft model UV-induced lesion formation	Spontaneous tumor formation	UV DNA damage induced tumor formation	No cSCC formation Chemically-induced carcinogenesis in digestive tract	Spontaneous tumor formation

*This table compares the impact of several β-HPVs and high risk α-HPVs on DDR and other pathways relevant to carcinogenesis. ↑ and ↓ indicate increased or decreased abundance, respectively*.

## Author contributions

Wrote the manuscript: SW and NW. Reviewed and edited the manuscript: SW and NW.

### Conflict of interest statement

The authors declare that the research was conducted in the absence of any commercial or financial relationships that could be construed as a potential conflict of interest.
